# Laparoscopic liver biopsy in the diagnosis of hepatic epithelioid hemangioendothelioma: A case report

**DOI:** 10.3892/ol.2014.2308

**Published:** 2014-07-02

**Authors:** YILEI DENG, YONG ZHOU, NANSHENG CHENG

**Affiliations:** Department of Biliary Surgery, West China Hospital, Sichuan University, Chengdu, Sichuan 610041, P.R. China

**Keywords:** laparoscopy, hepatic epithelioid hemangioendothelioma, liver biopsy

## Abstract

Hepatic epithelioid hemangioendothelioma (HEH) is a rare vascular tumor of the liver, and its definitive diagnosis is completely dependent on histopathological verification. In the present study, we report the case of a patient whose percutaneous liver biopsy failed to reveal a diagnosis of HEH, twice, and who was ultimately diagnosed by laparoscopic liver biopsy. The patient was a 42-year-old female with mild right upper quadrant discomfort. Ultrasonography and magnetic resonance imaging showed multiple mass lesions scattered throughout the liver, but no evidence of extrahepatic diseases. The initial laboratory tests included liver function tests and tumor markers were within normal limits. Subsequently, two, ultrasound (US)-guided liver biopsies from the liver lesion were performed using an 18-gauge needle, and both of these showed massive hepatocellular necrosis. To obtain adequate tissue samples for histological examination, the patient underwent laparoscopic liver biopsy. The overall immunohistochemical findings supported the diagnosis of HEH. In the present case, two, US-guided percutaneous liver biopsies failed to diagnose HEH, and laparoscopic liver biopsy was safely performed to obtained adequate specimens for analysis. Although this method is not the preferred technique and has certain disadvantages, it is considered to be a useful and minimally invasive approach for liver lesions when other less-invasive diagnostic modalities fail or are difficult to be performed.

## Introduction

Hepatic epithelioid hemangioendothelioma (HEH) is a rare vascular tumor of the liver with low- to intermediate-grade malignancy. Due to the rare tumor incidence and non-specific clinical presentation, HEH is difficult to differentiate from focal liver lesions including hepatocellular carcinoma, angiosarcoma and metastatic carcinoma (1–3). Although modern cross-sectional imaging techniques display the typical radiographic features of HEH, such as coalescence of nodules, capsular retraction and intralesional calcifications, the final definitive diagnosis of HEH is entirely dependent on histopathological verification (1–3).

In general, fine needle biopsy is able to provide a valuable diagnosis of HEH, but this can be confused with other conditions, particularly sclerosing hemangioma and sclerosing adenocarcinoma (1). In patients with HEH, parenchymal abnormalities are irregularly distributed and sampling variability is almost inevitable. Therefore, the specificity of the diagnosis mainly depends on precise tumor localization and a biopsy specimen of sufficient size (4). In the present study, we report the case of a patient whose underwent two, percutaneous liver biopsies, both of which failed to reveal a diagnosis of HEH. The patient was ultimately diagnosed by laparoscopic liver biopsy, as this technique allowed adequate tissue sampling under direct vision. The patient provided written informed consent.

## Case report

A 42-year-old female was admitted to West China Hospital, Sichuan University (Chengdu, China) in February, 2013 with mild right upper quadrant discomfort. The patient had no history of drug and alcohol abuse, or hepatitis. An abdominal ultrasound and the initial laboratory tests were scheduled. Ultrasonography showed multiple hypoechoic masses in the liver, whereas the liver function tests were in the normal range. Tumor markers, including α-fetoprotein (AFP), cancer embryonic antigen (CEA), carbohydrate antigen 199 (CA199), and CA125 were all within normal limits.

**Figure 1:**
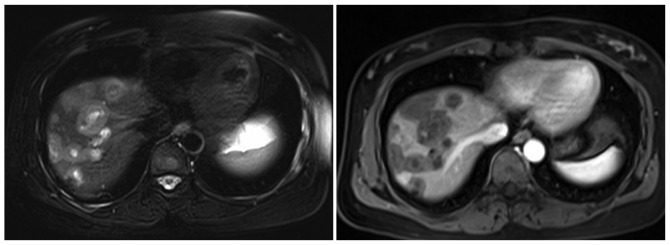
Magnetic resonance imaging of the liver. Left: T2-weighted image showing multiple high-signal intensity mass lesions scattered throughout the liver. Right: T1-weighted image showing multiple low-signal intensity mass lesions.

In view of such findings, liver metastases were suspected and, thus, whole body magnetic resonance imaging (MRI) was performed to find the primary tumor. MRI showed multiple mass lesions scattered throughout the liver with low-signal intensity on T1-weighted imaging (T1WI) and high-signal intensity on T2-weighted imaging (T2WI) (Fig. 1). There was no evidence of extrahepatic disease. Subsequently, ultrasound (US)-guided liver biopsy from the largest liver lesion was performed using an 18-gauge needle. This showed massive hepatocellular necrosis mixed with some epithelial cells. As the first biopsy was considered to have sampling error, a repeat biopsy was performed from the second largest liver lesion and the specimen was analyzed by the senior pathologists. However, the results still showed massive hepatocellular necrosis and no obvious atypical epithelial proliferation. Following consultation with the pathologists, inadequate specimen (1 mm in diameter and <2 cm in length) was considered to be the main reason for diagnosis failure. Therefore, laparoscopic liver biopsy was performed to obtain adequate tissue samples for histological examination.

**Figure 2:**
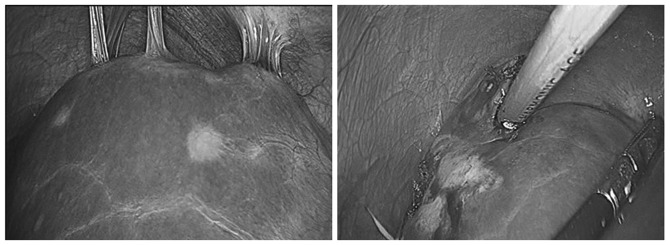
Intraoperative imaging of laparoscopic liver biopsy.

Under general anesthesia, carbon dioxide pneumoperitoneum was achieved with the patient in supine position. Three laparoscopic ports were inserted: A 10-mm camera port was placed immediately below the umbilicus, a 12-mm trocar was placed below the xiphoid process and a 5-mm trocar was placed below the rib cage at the level of the right midclavicular line. Several masses of varying size and with a gray appearance were seen protruding from the surface of the right liver lobe (Fig. 2). A wedge resection liver mass biopsy of ~1.5×1.5×1.0 cm in size was obtained using an ultrasonic scalpel (Harmonic Scalpel; Ethicon Endo-Surgery Inc., Cincinnati, OH, USA) (Fig. 2). The patient was discharged on the first postoperative day with no complications. After one week, the histopathology report revealed medium -to large-sized pleiomorphic cells spread within the sinusoids and small veins. These cells stained positive for CD31, CD34 and factor VIII-related antigen, as well as CK7, CK19 and phosphoenolpyruvate carboxykinase. Thus, the overall immunohistochemical findings supported the diagnosis of HEH. Therefore, we recommended that the patient undergo liver transplantation, but the patient refused. During the past eight months of follow-up after discharge, the patient has been asymptomatic and liver ultrasonography at two-month intervals has shown no significant change with respect to lesion size.

## Discussion

HEH is a rare tumor with an incidence of <0.1 per 100,000 individuals worldwide (5). The clinical manifestation of HEH varies from asymptomatic to non-specific symptoms, such as right upper quadrant discomfort, weight loss and abnormal liver function (3). In addition, routine laboratory tests are usually inconclusive and normal serum tumor markers, including AFP, CEA, CA199 and CA125, do not exclude other primary and secondary liver tumors. In the majority of patients, the tumor is first discovered incidentally during imaging studies. Although typical imaging features of HEH, such as coalescence of nodules, capsular retraction and intralesional calcifications, have been proposed to be useful in improving the diagnosis of this rare hepatic tumor, great imaging heterogeneity still persists (2). Additionally, definitive diagnosis of HEH requires histopathological examination. Diagnosis of HEH is mostly confirmed by immunohistochemical evidence of endothelial differentiation, which mainly depends on the detection of the expression of certain key endothelial cell markers, such as CD31, CD34 and factor VIII-related antigen (1). In the present case, a diagnosis of HEH was missed in the two core biopsies, which may have been due to poor biopsy specimens that were insufficient in size and with central necrosis in the liver mass.

In hepatic tumors such as HEH, parenchymal abnormalities are irregularly distributed and sampling variability is almost inevitable (4). Thus, in order to improve the accuracy of diagnosis and further grading and tumor staging, the most practical solution appears to be obtaining a biopsy specimen of sufficient size. Laparoscopic liver biopsy not only allows for the systematic visualization of lesions, but also obtains adequate tissue samples under direct vision (4,5). However, according to the various related literature, despite a high sensitivity and specificity of laparoscopic liver biopsy for the diagnosis of liver lesions, the diagnostic accuracy varies for different disease process: 98% for chronic liver disease, 91% for abnormal liver function and only 85% for cancer (6). In addition, complications of laparoscopic liver biopsy include general anesthesia, bowel perforation, bleeding from the biopsy site and local abdominal wall trauma (5,7). Additionally, high expenses and the requirement for special expertise in performing the procedure have limited its use. However, biopsies performed with narrower gauge needles (smaller than 18 gauge) have occasionally been found to be adequate to establish the diagnosis (4). To the best of our knowledge, there are no direct comparisons of the complications and outcomes between percutaneous and laparoscopic biopsy to date. However, it should be noted that use of a thin biopsy needle may increase the sampling error and lead to an incorrect diagnosis, due to an insufficient sample size (8).

In summary, in the present case, when US-guided percutaneous liver biopsy failed to diagnose HEH, laparoscopic liver biopsy was safely performed, obtaining adequate specimens for analysis. Although this method was not the preferred technique and has certain drawbacks, it is considered to be a useful and minimally invasive approach for liver lesions when other less-invasive diagnostic modalities fail or are difficult to be performed.
